# Astaxanthin protects against osteoarthritis via Nrf2: a guardian of cartilage homeostasis

**DOI:** 10.18632/aging.102474

**Published:** 2019-11-26

**Authors:** Kai Sun, Jiahui Luo, Xingzhi Jing, Jiachao Guo, Xudong Yao, Xiaoxia Hao, Yaping Ye, Shuang Liang, Jiamin Lin, Genchun Wang, Fengjing Guo

**Affiliations:** 1 Department of Orthopedics, Tongji Hospital, Tongji Medical College, Huazhong University of Science and Technology, Wuhan 430030, China; 2 The Center for Biomedical Research, Ministry of Education and Ministry of Health, Tongji Hospital, Tongji Medical College, Huazhong University of Science and Technology, Wuhan 430030, China; 3 Department of Rehabilitation, Tongji Hospital, Tongji Medical College, Huazhong University of Science and Technology, Wuhan 430030, China

**Keywords:** osteoarthritis, cartilage homeostasis, astaxanthin, Nrf2, apoptosis

## Abstract

Scope: Osteoarthritis (OA) is a progressive disease characterized by cartilage degradation. Astaxanthin (Ast), a natural compound with remarkable antioxidant activity and multiple medical applications due to its activation of Nrf2 signaling, has been studied for application to various degenerative diseases. Currently, however, little is known about its efficacy in treating OA. This study reports the effects of Ast on cartilage homeostasis in OA progression.

Methods: IL-1β, TNF-α, and tert-butyl hydroperoxide (TBHP) were used to impair cartilage homeostasis. Modulating effects of Ast on the Nrf2 signaling pathway, and damage-associated events including extracellular matrix (ECM) degradation, inflammation, oxidative stress, chondrocyte apoptosis, and *in vivo* cartilage degradation were examined.

Results: Ast attenuated ECM degradation of OA chondrocytes through the Nrf2 signaling, and ameliorated the IL-1β-induced inflammatory response and ECM degradation via blockade of MAPK signaling. Additionally, Ast alleviated TNF-α-induced ECM degradation and chondrocyte apoptosis by inhibiting the NF-κB signaling, suppressed TBHP-induced oxidative stress, and subsequently reduced chondrocyte apoptosis. *In vitro* results were finally corroborated *in vivo* by demonstrating that Ast attenuates the severity of cartilage destruction in a mouse model of OA.

Conclusions: Ast could protect against osteoarthritis via the Nrf2 signaling, suggesting Ast might be a potential therapeutic supplement for OA treatment.

## INTRODUCTION

Osteoarthritis (OA), a common degenerative disorder affecting more than 240 million people, is a primary cause of disability [1]. The disease is strongly age-dependent, and is characterized by increased destructive alteration of articular cartilage, synovial tissue, and subchondral bone, as well as osteophyte formation [2]. Aging is not the sole risk factor for OA progression. The pathogenesis is complex, and involves physiological and mechanical factors including sex, obesity, heredity, metabolic alterations, trauma, and mechanical stress [3], each of which can affect all tissues of the articular joint. Cartilage degradation, caused by an imbalance in cartilage homeostasis, is a central feature of OA [4]. Various factors activate chondrocytes to promote extracellular matrix degradation. Once cartilage homeostasis is disrupted, damage-associated signals will be transduced and amplified by a feed-forward loop, which ultimately leads to degradation and loss of cartilage [4]. Many of these OA factors can also induce low-grade chronic inflammation, and imbalance in oxidant–antioxidant levels, stimulating chondrocytes to produce reactive oxygen species (ROS) and pro-inflammatory cytokines including IL-1β, and TNF-α. These mediators are also second messengers in intracellular signaling pathways that regulate the expression of the target genes encoding matrix degrading enzymes. Overproduction of IL-1β, TNF-α, and H_2_O_2_ by activated synoviocytes, macrophages, and chondrocytes, plays a role in the destruction of cartilage homeostasis [5]. IL-1β is known to trigger the production and secretion of inflammatory mediators, which contribute to OA pathogenesis [6]. TNF-α, another vital pro-inflammatory cytokine linked to OA, could disturb catabolic and anabolic pathways in chondrocytes. In addition, TNF-α plays an important role in inducing ROS production and cell apoptosis [6]. Hydrogen peroxide (H_2_O_2_), a common species of ROS, is harmful to normal cells, including chondrocytes, if overproduced [7]. Oxidative stress caused by H_2_O_2_ could induce ECM degradation and result in chondrocyte apoptosis [8]. These mediators have overlapping effects, but each has its own role in mediating OA pathogenesis. Therefore, it is relevant to use these mediators to induce OA *in vitro* to explore the pathogenesis of OA, and investigate possible therapeutic approaches.

NF-E2-related nuclear factor 2 (Nrf2) is the master sensor of oxidative stress, and a regulator of cellular redox homeostasis [9]. Nrf2 is liberated from its repressor Keap1, and subsequently regulates expression of various cytoprotective genes including heme oxygenase-1 (HO-1) and NADPH quinone oxidoreductase1 (NQO1) on exposure to stresses [9]. Nrf2 signaling pathway activators have been demonstrated to provide multiple protective effects in experimental models of chronic diseases including diabetes, cardiac disease, and neurodegenerative diseases [10]. Evidence supporting an essential role of Nrf2 in OA progression has recently begun to accumulate. Nrf2 is a stress response regulator that exerts anti-oxidative and anti-inflammatory effects in OA chondrocytes [11, 12]. Therefore, it is important to investigate the protective effects of Nrf2 on OA pathogenesis.

Astaxanthin (Ast), known as a “marine carotenoid”, is widely present in aquatic animals including shrimp, lobster, salmon, trout, red seabream, and fish eggs [13]. Ast is a keto-carotenoid with antioxidant effects 100 times more potent than canthaxanthin and β-carotene [14]. It shows auspicious effects on human health, with excellent safety and tolerability. Various important biological activities of Ast, and potentially beneficial effects in various diseases have been highlighted and are discussed in the present research, including inflammatory diseases, skin diseases, obesity, cancer, and cardiovascular diseases. Some of these studies have shown that Ast suppresses inflammation and oxidative stress in macrophages via Nrf2 [15]. Ast also exerts inhibitory effects on oxidative stress and apoptosis of hematopoietic progenitor cells through activation of Nrf2/HO-1 [16]. With regard to OA, previous studies have reported that Ast reduces IL-1β-induced MMP expression in chondrocytes, and ameliorates cartilage loss in experimental osteoarthritis [17, 18]. Based on these findings, we hypothesized that Ast might facilitate cartilage homeostasis under various harmful conditions, and attenuate progression of OA via Nrf2-mediated protective effects.

Due to its powerful bioactivity and its safety, Ast has been approved by the FDA as a food additive, and is widely used as a nutraceutical by athletes [13, 19]. The effect of Ast on reducing matrix metalloproteinase expression has been described previously. However, other beneficial effects of Ast on OA progression remain unclear, such as anti-inflammatory, anti-oxidant, and anti-apoptotic effects. Furthermore, how Nrf2-mediated regulation, and other molecular mechanisms facilitate cartilage homeostasis have yet to be determined. In the present study, we sought to explore the effects of Ast on OA chondrocytes and cartilage, and the regulatory effects of the Nrf2 signaling pathway.

## RESULTS

### Ast did not affect chondrocyte viability

**Figure 1:**
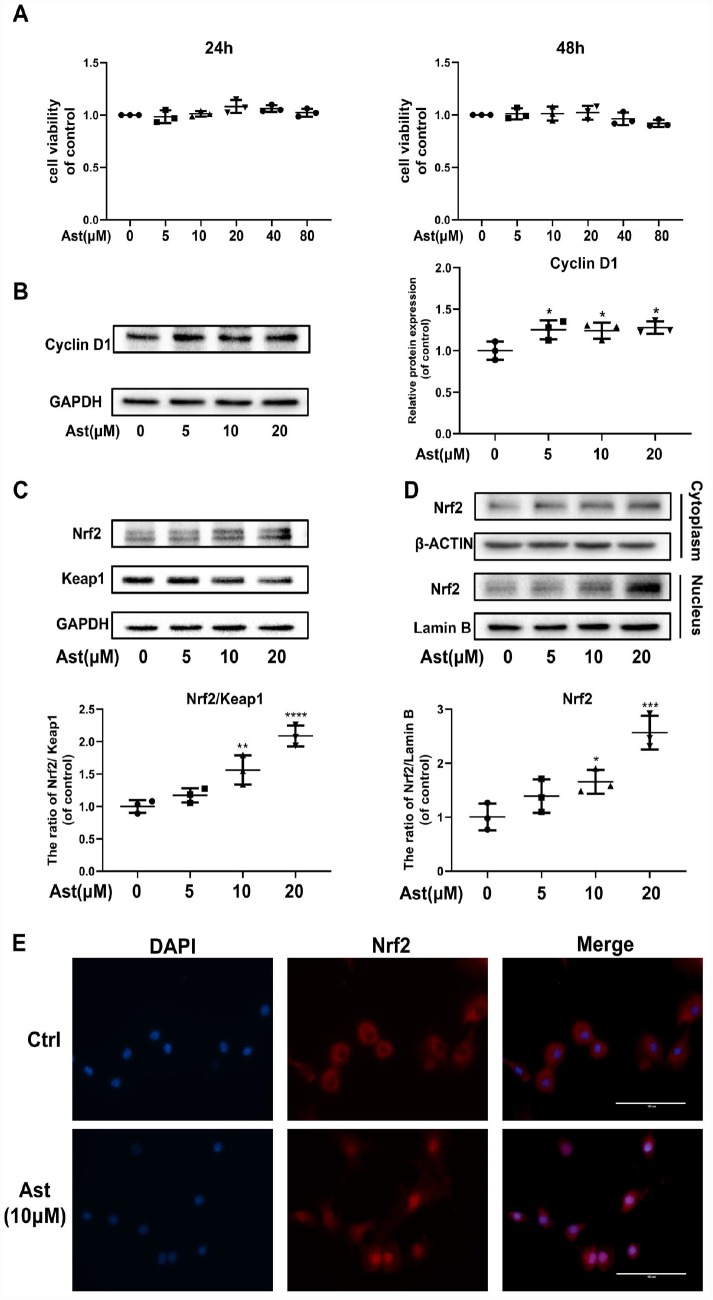
**Ast did not affect cell viability and activated Nrf2 in mouse chondrocytes.** (**A**) The cytotoxic effect of Ast (5, 10, 20, 40, and 80 μM) exposure for 24 and 48 h on chondrocytes was determined using a CCK8 assay. (**B**, **C**) Chondrocytes were treated with Ast (5, 10, and 20 μM) for 24 h. Expression levels of Cyclin D1, Nrf2, and Keap1 were determined by western blotting and quantified. (**D**, **E**) Nuclear translocation of Nrf2 was detected by western blotting and immunofluorescence after treatment of chondrocytes with Ast (10 μM) for 24 h, and the band density of Nrf2 in nucleus was quantified. The nuclear and cytoplasmic fractions used in the western blotting were obtained using a nuclear and cytoplasmic protein extraction kit (P0027, Beyotime, China). The data are presented as dot plots from three independent experiments. Significant differences among different groups are indicated as *p < 0.05 vs. control; **p<0.01 vs. control; ****p<0.0001 vs. control.

The cytotoxic effects of Ast on mouse chondrocytes were determined at various concentrations (5, 10, 20, 40, and 80 μM) for 24 h and 48 h (Figure 1A). These concentrations of Ast did not affect cell viability. Therefore, 5, 10, and 20 μM Ast were utilized for subsequent experiments. We examined the effect of Ast on the chondrocyte proliferation. Ast (5, 10, and 20 μM) upregulated the level of Cyclin D1 protein (Figure 1B), indicating that Ast could promote proliferation of chondrocytes.

### Ast activated the Nrf2 transcription factor in chondrocytes

Since Ast exerts powerful anti-oxidant effects, and the transcription factor Nrf2 plays a vital role in redox balance in cellular homeostasis, we hypothesized that Ast treatment might activate the Nrf2 signaling pathway. Therefore, we measured the Nrf2/Keap1 system in Ast-treated chondrocytes by measuring protein levels and nuclear translocation of Nrf2. After Ast (5, 10, and 20 μM) treatment, a remarkable dose-dependent upregulation of Nrf2, with corresponding decline in expression of Keap1 was seen (Figure 1C). Ast treatment also led to significant nuclear translocation of Nrf2 (Figure 1D, 1E). These results indicate that Ast could activate Nrf2 by preventing its sequestration by Keap1.

### The effects of OA inducers IL-1β, TNF-α, and TBHP on chondrocytes

**Figure 2:**
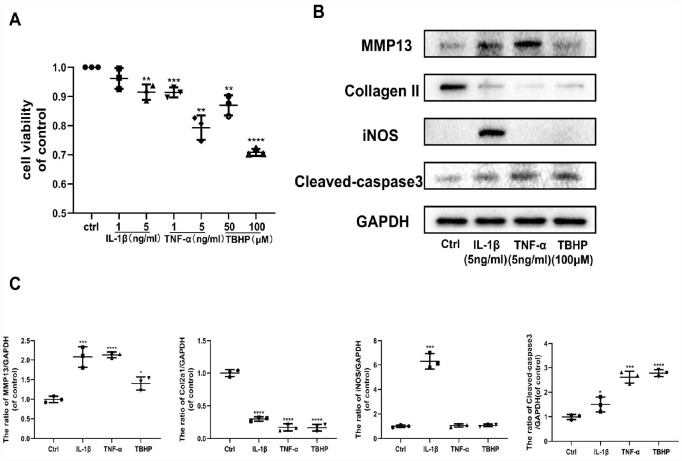
**Effects of IL-1β, TNF-α, and TBHP on mouse chondrocytes.** (**A**) The cytotoxic effect of IL-1β (1 and 5 ng/ml), TNF-α (1 and 5 ng/ml), and TBHP (50 and 100 μM) treatment on chondrocytes for 24 h was determined using a CCK8 assay. (**B**, **C**) After the indicated treatment for 24 h, the effects of IL-1β (5 ng/ml), TNF-α (5 ng/ml), and TBHP (100 μM) on the expression of MMP13, Collagen II, iNOS, and cleaved-caspase3 were determined by western blotting and quantified. The data are presented as dot plots from three independent experiments. Significant differences among different groups are indicated as *p < 0.05 vs. control; **p<0.01 vs. control; ***p<0.001 vs. control; ****p<0.0001 vs. control.

The pathological progression of OA is complex and multifactorial. Various factors stimulate cells in the articular cavity to overproduce multiple mediators, primarily IL-1β, TNF-α, and H_2_O_2_, promoting OA development. Therefore, we assessed the impact of IL-1β, TNF-α, and TBHP (an organic hydrogen peroxide far more stable than H_2_O_2_) separately on chondrocytes by studying their effects on specific target protein levels. First, IL-1β, TNF-α, and TBHP affected chondrocyte viability in a dose-independent manner (Figure 2A). Thus, we chose effective concentrations of IL-1β (5 ng/ml), TNF-α (5 ng/ml) and TBHP (100 μM) for the next experiment. IL-1β, TNF-α, and TBHP disrupted cartilage homeostasis by upregulating expression of MMP13, and downregulating Collagen II in protein levels. In addition, IL-1β exerted a stronger pro-inflammatory effect on chondrocytes than TNF-α and TBHP by promoting expression of iNOS. By contrast, TNF-α and TBHP induced obvious chondrocyte apoptosis by increasing expression of cleaved-caspase 3 protein (Figure 2B, 2C). All these results indicate that OA inducers, including IL-1β, TNF-α, and H_2_O_2_ can promote progression of osteoarthritis in different ways. Therefore, we examined the role of Ast on OA progression under these pathological conditions.

### Ast safeguarded cartilage homeostasis under pathological conditions

**Figure 3:**
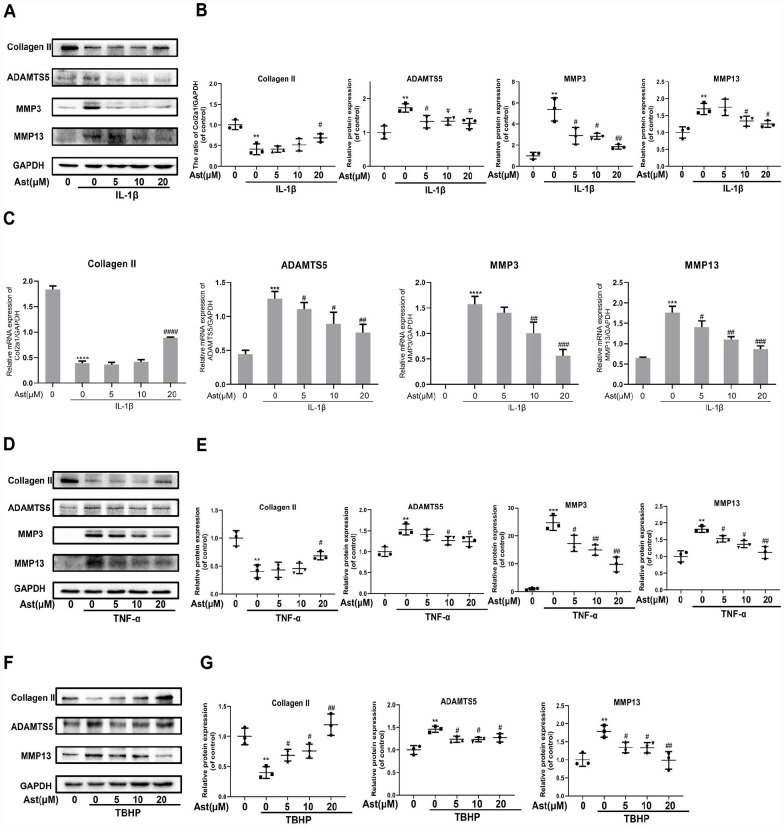
**Effects of Ast on OA chondrocytes.** Chondrocytes were pretreated with or without Ast (5, 10, and 20 μM) for 2 h and then exposed to IL-1β (5 ng/ml) for 24 h. Then, (**A**–**C**) the expression of Collagen II, ADAMTS5, MMP3, and MMP13 was examined by RT-PCR and western blotting and quantified. Chondrocytes were treated with the vehicle or with increasing concentrations of Ast for 2 h followed by (5 ng/ml) TNF-α stimulation. (**D**, **E**) The expression of Collagen II, ADAMTS5, MMP3, and MMP13 were determined by western blotting and quantified. After the cells were treated with the vehicle or Ast (5, 10, and 20 μM) for 2 h, and stimulated with 100 μM TBHP, (**F**, **G**) the expression of Collagen II, ADAMTS5, and MMP13 was investigated by western blotting and quantified. The data are presented as dot plots from three independent experiments. Significant differences among different groups are indicated as **p<0.01 vs. control; ***p<0.001 vs. control; ****p<0.0001 vs. control. #p < 0.05 vs. IL-1β or TNF-α or TBHP group; ##p < 0.01 vs. IL-1β or TNF-α or TBHP group; ###p < 0.001 vs. IL-1β or TNF-α or TBHP group; ####p < 0.0001 vs. IL-1β or TNF-α or TBHP group.

In response to stimulation with IL-1β, TNF-α, and H_2_O_2_, chondrocytes produce ADAMTS5 and MMPs, and reduce expression of Collagen II. These effects contribute to OA pathogenesis [20–23]. We investigated whether Ast could maintain cartilage homeostasis under these pathological conditions. Our results showed that IL-1β caused notably increased mRNA levels of ADAMTS5, MMP3, and MMP-13, and decreased expression of Collagen II. Stimulation by IL-1β, TNF-α, and TBHP resulted in obvious downregulation of Collagen II and upregulation of ADAMTS5 and MMP protein levels. Ast treatment was found to markedly reverse these changes (Figure 3A–3G). These results indicate that Ast could effectively safeguard cartilage homeostasis in various pathological environments.

### Ast activated Nrf2 to maintain cartilage homeostasis under pathological conditions

**Figure 4:**
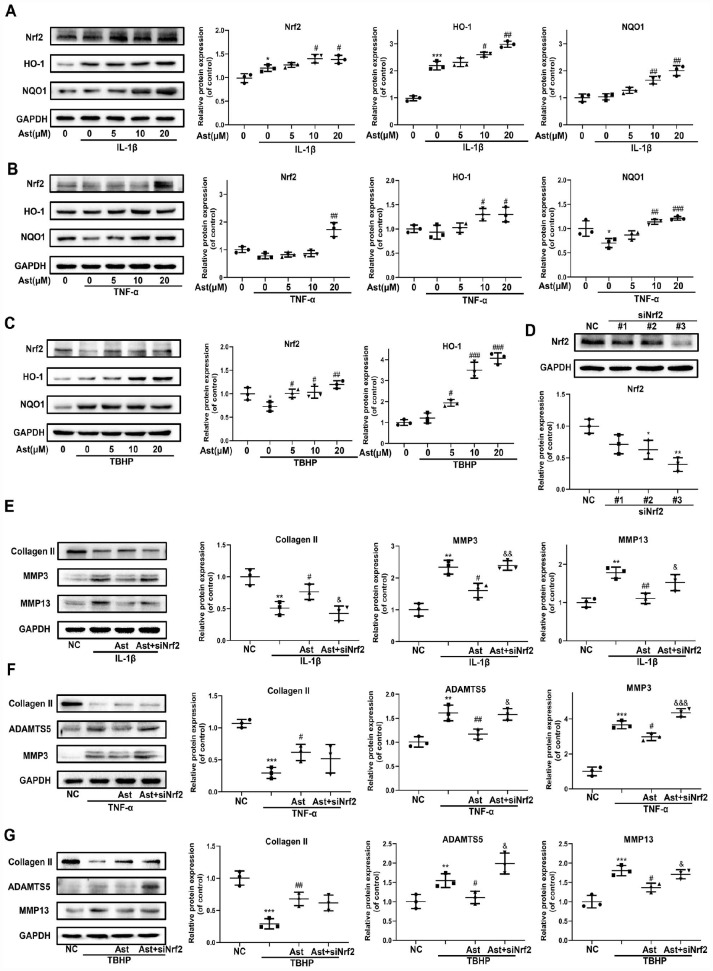
**Nrf2 signaling mediated protective effects of Ast on OA chondrocytes.** Chondrocytes were cultured with vehicle or Ast (5, 10 and 20 μM) for 2 h, then stimulated with IL-1β (5 ng/ml), TNF-α (5 ng/ml) or TBHP (100 μM). (**A**–**C**) Levels of Nrf2 signaling pathway proteins, including Nrf2, HO-1, and NQO1 were determined by western blotting and quantified. To knock down Nrf2 expression, chondrocytes were transfected with Nrf2 siRNA using Lipofectamine 3000. (**D**) Transfection efficiency was evaluated by detecting Nrf2 expression using western blotting. After 24h of transfection, chondrocytes were treated with vehicle or Ast (10 μM) for 2 h followed by stimulation with IL-1β (5 ng/ml) for 24 h. (**E**, **F**) The expression of Collagen II, ADAMTS5, MMP3, and MMP13 was measured by western blotting and quantified. The data are presented as dot plots from three independent experiments. Significant differences among different groups are indicated as *p<0.05 vs. control; **p<0.01 vs. control; ***p<0.001 vs. control; ****p<0.0001 vs. control. #p < 0.05 vs. IL-1β or TNF-α or TBHP group; ##p < 0.01 vs. IL-1β or TNF-α or TBHP group; ###p < 0.001 vs. IL-1β or TNF-α or TBHP group; ####p < 0.0001 vs. IL-1β or TNF-α or TBHP group. &p < 0.05 vs. (IL-1β or TNF-α or TBHP) + Ast group; &&p < 0.01 vs. (IL-1β or TNF-α or TBHP) + Ast group; &&&p < 0.001 vs. (IL-1β or TNF-α or TBHP) + Ast group.

Cellular redox balance is indispensable to cartilage homeostasis. The Nrf2 anti-oxidant system acts as a master regulator of cellular redox balance. Ast exerts various cytoprotective effects by promoting Nrf2/ARE signaling [15, 24]. Therefore, we investigated whether Ast could activate the Nrf2 system in OA chondrocytes. Ast treatment led to notably increased protein levels of total Nrf2 and downstream cytoprotective genes including HO-1 and NQO1 in mouse chondrocytes stimulated with IL-1β, TNF-α, and TBHP (Figure 4A–4C). To investigate whether Nrf2 plays protective roles in cartilage homeostasis in OA chondrocytes, Nrf2 knockdown was performed by siRNA transfection (Figure 4D #3). We then examined changes in expression of ADAMTS5, MMPs, and Collagen II after cells were treated as in Figure 4E–4G. Knockdown of Nrf2 abrogated Ast-mediated inhibition of IL-1β, TNF- α, and TBHP-induced expression of ADAMTS5 and MMPs in chondrocytes. In addition, Nrf2 knockdown abolished Ast-mediated upregulation of Collagen II in IL-1β-induced chondrocytes. These data indicate that Nrf2 may at least partially protect cartilage homeostasis against damage due to IL-1β, TNF-α and TBHP.

### Ast modulated an IL-1β-induced inflammatory response, and ECM degradation via the MAPK signaling pathway

Previous studies have shown that inflammatory synovitis is always associated with OA, and causes inflammation-related symptoms [25]. OA chondrocytes and synovial cells overproduce inflammatory mediators such as IL-1β and nitric oxide to accelerate cartilage degradation [6]. IL-1β can also markedly induce inflammatory reactions (Figure 1B). Therefore, we investigated whether Ast can exert anti-inflammatory effects on IL-1β-stimulated chondrocytes.

**Figure 5:**
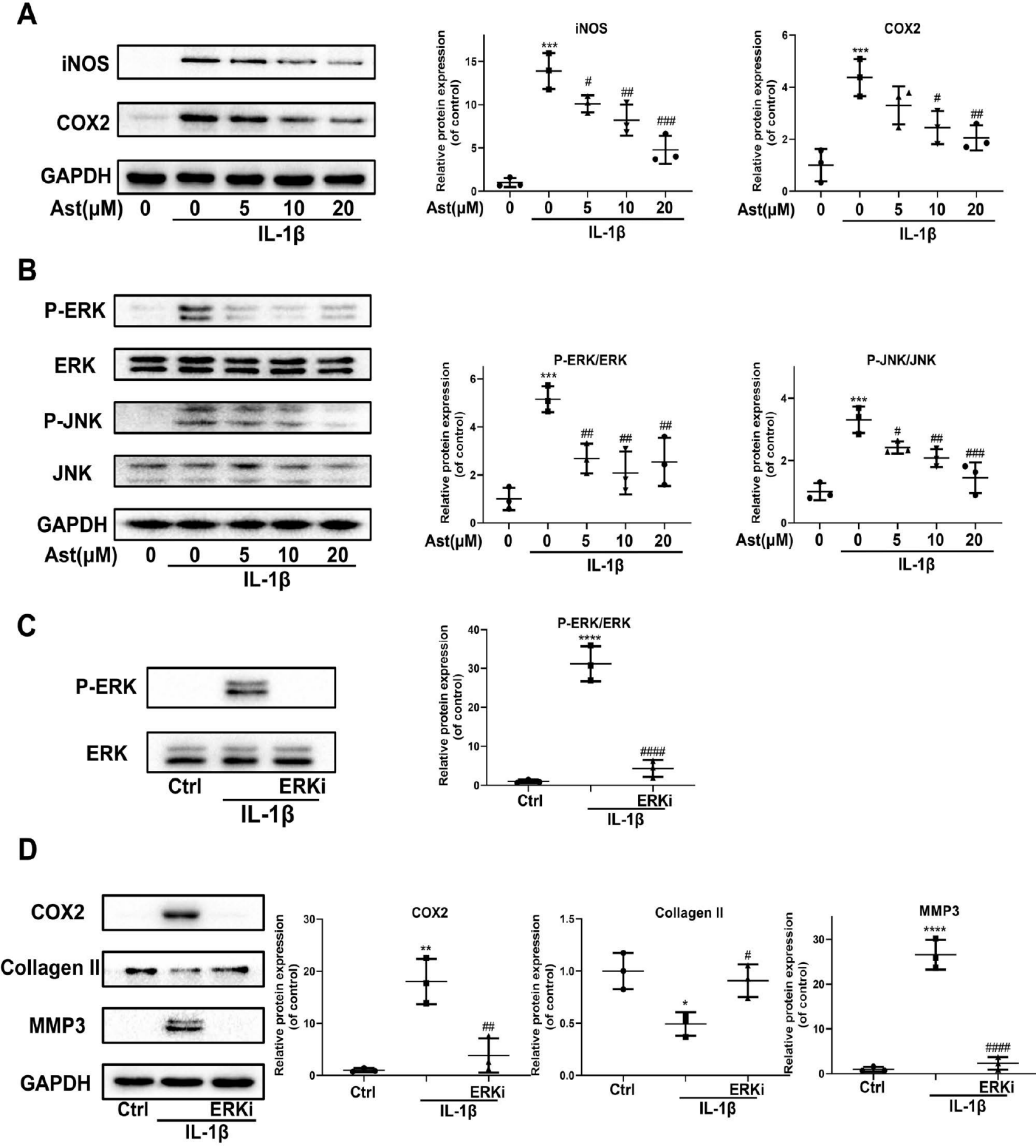
**Effects of Ast on IL-1β-induced inflammatory response.** Chondrocytes were treated with Ast (5, 10, and 20 μM) for 2 h followed by stimulation with vehicle or IL-1β (5 ng/mL) for 24 h. (**A**) Expression of iNOS and COX-2 protein assessed by western blotting and quantified. (**B**) To detect altered phosphorylation within the MAPK signaling pathway, chondrocytes were serum-starved for 6 h followed by treatment with vehicle or Ast (5,10, and 20 μM) for 2 h. Cells were then stimulated with IL-1β (5 ng/mL) for 30 min. MAPK activation was examined using western blotting and quantified. (**C**) Phosphorylation of ERK was detected by western blotting after chondrocytes were pre-treated with vehicle or PD0325901 (a MEK inhibitor) for 2 h, followed by stimulation with IL-1β (5 ng/ml). (**D**) Chondrocytes were treated as indicated for 24 h. The expression of COX2, Collagen II, and MMP3 was determined using western blotting and quantified. The data are presented as dot plots from three independent experiments. Significant differences among different groups are indicated as *p<0.05 vs. control; **p<0.01 vs. control; ***p<0.001 vs. control; ****p<0.0001 vs. control. #p < 0.05 vs. IL-1β group; ##p < 0.01 vs. IL-1β group; ###p < 0.001 vs. IL-1β group; ####p < 0.0001 vs. IL-1β group.

Our results showed that Ast reduced IL-1β-induced upregulation of iNOS and COX2 protein levels (Figure 5A). In addition, Ast blocked IL-1β-induced phosphorylation of ERK and JNK (Figure 5B). We hypothesized that the MAPK signaling pathway may be involved in Ast-mediated suppression of IL-1β-induced inflammation, and in ECM degradation. Our data showed that an ERK inhibitor can reverse IL-1β-induced phosphorylation of ERK, and can further inhibit the increased expression of COX2 and MMP3, and decreased expression of Collagen II, similar to the effects of Ast treatment (Figure 5C, 5D). These results indirectly demonstrate that Ast can modulate the IL-1β-induced inflammatory response, and cartilage homeostasis via the MAPK signaling pathway.

### Ast reduced TNF-α-induced apoptosis and ECM degradation in chondrocytes via the NF-κB signaling pathway

**Figure 6:**
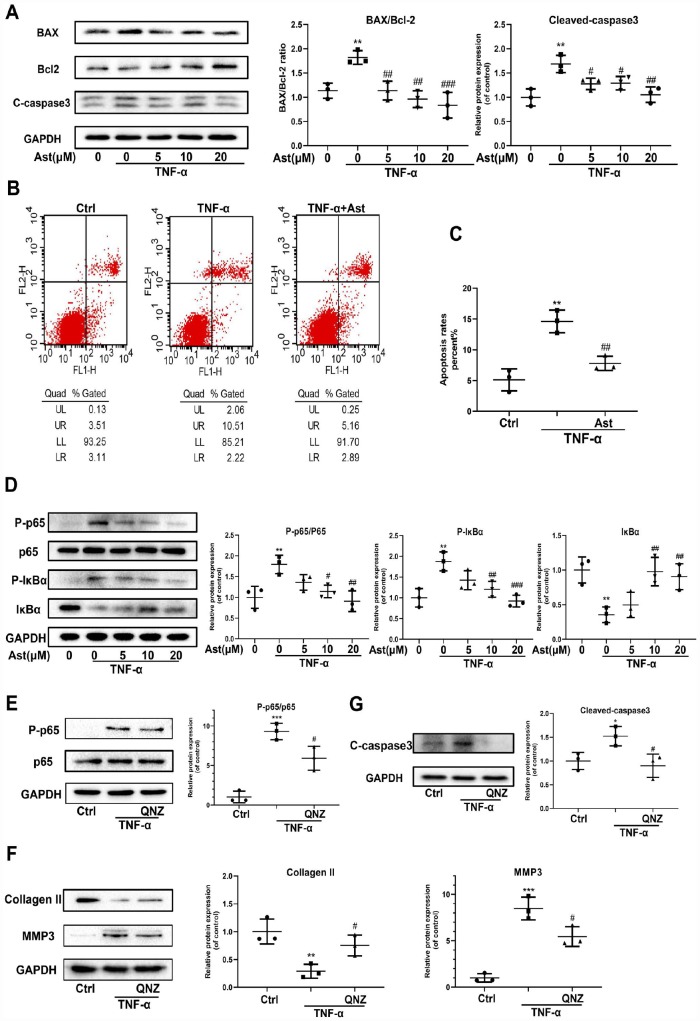
**Effects of Ast on TNF-α-induced apoptosis and ECM degradation.** (**A**, **B**) Protein levels of BAX, Bcl-2, and cleaved caspase-3 were determined by western blotting, and apoptotic chondrocytes were stained with Annexin V-FITC/PI and examined by flow cytometry after treated as above for 24 h. Apoptosis rate was calculated and the data were expressed in (**C**). FL1 represents Annexin V-FITC and FL2 represents PI. To detect the activation of NF-κB signaling, chondrocytes were serum-starved for 6 h followed by treatment with the vehicle or Ast (5,10, and 20 μM) for 2 h. Cells were then stimulated with TNF-α (5 ng/ml) for 15 min. (**D**) Activation of the NF-κB signaling pathway was measured by western blotting and quantified. (**E**) Phosphorylation of p65 was detected by western blotting after the chondrocytes were pre-treated with the vehicle or QNZ (an inhibitor of the NF-κB pathway) for 2 h, followed by stimulation with TNF-α (5 ng/ml). (**F**, **G**) Chondrocytes were treated as indicated for 24 h. The expression of Collagen II, MMP3 and cleaved-caspase3 was determined using western blotting and quantified. The data are presented as dot plots from three independent experiments. Significant differences among different groups are indicated as *p<0.05 vs. control; **p<0.01 vs. control; ***p<0.001 vs. control. #p < 0.05 vs. TNF-α group; ##p < 0.01 vs. TNF-α group; ###p < 0.001 vs. TNF-α group.

TNF-α, another critical pro-inflammatory mediator, could drive chondrocyte death and thereby destroy cartilage homeostasis [26, 27]. NF-κB mediates a TNF-α-related signaling pathway, regulating cellular events including proliferation, hypertrophy, and apoptosis [28]. Ast can suppress LPS-stimulated NF-kB activation [29]. Therefore, we investigated whether Ast could reduce TNF-α-induced apoptosis of chondrocytes, and ECM degradation via the NF-κB signaling pathway. The results showed BAX and cleaved-caspase 3 protein levels were increased in TNF-α-stimulated chondrocytes, whereas expression of Bcl-2 protein was decreased. These trends were reversed by pretreatment with Ast (Figure 6A). We performed flow cytometric analysis to assess chondrocyte apoptotic rates using Annexin V-FITC/PI staining. Stimulation of TNF-α increased chondrocyte apoptosis compared with the control group, while Ast supplementation significantly reduced TNF-α-induced apoptosis (Figure 6B, 6C). Furthermore, Ast inhibited TNF-α-induced phosphorylation of p65 and IκBα, and attenuated IκBα degradation (Figure 6D). To further verify the roles of NF-κB, the NF-κB inhibitor QNZ was used. Our data showed that QNZ can reverse the TNF-α-induced increases in cleaved-caspase3 and MMP3, and the decrease in Collagen II protein levels (Figure 6E–6G). These results were consistent with Ast treatment, which suggested that Ast can at least partially reduce TNF-α-induced apoptosis and ECM degradation via the NF-κB signaling pathway.

### Ast reduced TBHP-induced chondrocyte apoptosis by modulating redox homeostasis

**Figure 7:**
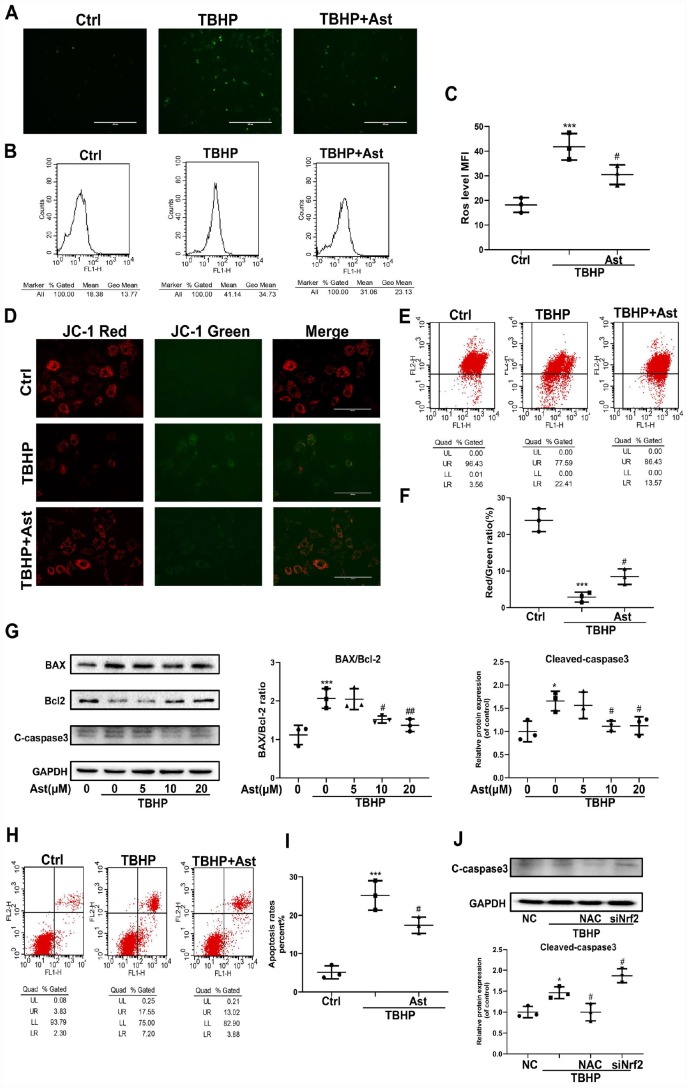
**Effects of Ast on TBHP-induced oxidative stress, and chondrocyte apoptosis.** Chondrocytes were treated with Ast (10 μM) for 2 h, then stimulated with TBHP (100 μM) for 24 h. The samples with the same concentration of chondrocytes were used to detect intracellular ROS. Intracellular ROS was stained by DCFH-DA followed by fluorescence spectrophotometry and flow cytometric analysis. (**A**) Green fluorescence indicates that the intracellular ROS production. (**B**) Representative data of flow cytometric measurement of ROS production. (**C**) Dot plots graphs show the mean fluorescence intensity (MFI) of ROS in chondrocytes. (**D**, **E**) Mitochondrial membrane potential (MMP) was detected by fluorescence and flow cytometric analysis after chondrocytes were incubated with JC-1. Red fluorescence represents JC-1 aggregates in healthy mitochondria, while green fluorescence is emitted by JC-1 monomers, representing MMP dissipation. Merged images exhibit co-localization of JC-1 aggregates and monomers. FL1 represents JC-1 green and FL2 represents JC-1 red. (**F**) Dot plots graphs represent flow cytometric analysis of MMP levels, represented as the ratio of red/green MFI. (**H**) To measure chondrocyte apoptosis, cells were stained with Annexin V-FITC/PI and apoptotic cells were counted by flow cytometry. FL1 represents Annexin V-FITC and FL2 represents PI. Apoptosis rates were evaluated, and the results are expressed as dot plots graphs (**I**). (**G**) Chondrocytes were treated with vehicle or Ast (5, 10, and 20 μM) for 2 h, then stimulated with TBHP (100 μM) for 24 h. Protein levels of BAX, Bcl-2, and cleaved-caspase3 were examined by western blotting and quantified. (**J**) Expression of cleaved-caspase 3 protein in chondrocytes under treatment with scrambled siRNA (NC), TBHP (100 μM), N-acetylcysteine (NAC)+TBHP, Nrf2-siRNA+TBHP, respectively. The data are presented as dot plots from three independent experiments. Significant differences among different groups are indicated as *p<0.05 vs. control; **p<0.01 vs. control; ***p<0.001 vs. control. #p < 0.05 vs. TBHP group; ##p < 0.01 vs. TBHP group.

H_2_O_2_ overload can trigger oxidative stress, with mitochondrial dysfunction via ROS overproduction and altered MMP levels, which further stimulates cell apoptosis [7]. Ast, a strong scavenger of ROS, has a prominent antioxidant capacity [30]. Therefore, we determined the effects of Ast on TBHP-induced oxidative stress and apoptosis in chondrocytes. DCFH-DA staining showed 100 μM TBHP triggered marked intracellular ROS production in chondrocytes. Ast treatment significantly suppressed green fluorescence intensity, representing a decrease in TBHP-induced elevated ROS production (Figure 7A). Flow cytometry was performed to quantify ROS generation, and this data exhibited a similar tendency (Figure 7B, 7C). Collapse of MMP means mitochondrial dysfunction and indicates an early stage of apoptosis. We determined whether Ast affects MMP using JC-1 staining and flow cytometry. The results showed that TBHP treatment resulted in obviously increased green fluorescence intensity of JC-1 monomers, rather than red fluorescence intensity, indicating that TBHP led to a reduction in MMP. Ast (10 μM) treatment reduced the elevation in green fluorescence intensity of JC-1 monomers induced by TBHP (Figure 7D). Flow cytometric analysis showed that Ast (10 μM) partly reversed the inhibitory effects of TBHP (100 μM) on the red fluorescence intensity of JC-1 aggregates (Figure 7E, 7F). These results suggested protective roles played by Ast on mitochondrial function. Finally, we examined whether Ast exerted its anti-apoptotic effects by modulating oxidative stress. Western blot results showed that Ast reversed TBHP-induced upregulation of BAX and cleaved-caspase 3, and downregulation of Bcl-2 protein levels (Figure 7G). Qualitative measurement of chondrocyte apoptosis by Annexin V-FITC/PI staining showed Ast (10 μM) treatment significantly decreased the apoptotic rate induced by TBHP (100 μM) (Figure 7H, 7I). To further ascertain that Ast inhibited TBHP-induced apoptosis of chondrocytes through modulation of oxidative stress, ROS generation was inhibited by N-acetylcysteine (NAC), and Nrf2 was knocked down to examine the change in TBHP-mediated apoptosis of chondrocytes. These results showed that NAC treatment reduced TBHP-induced expression of cleaved-caspase 3 protein, whereas knock down of Nrf2 increased TBHP-induced upregulation of cleaved-caspase 3 (Figure 7J), indirectly suggesting that Ast exerts anti-apoptotic effects via ROS scavenging and Nrf2 activation.

### Ast attenuated cartilage degradation *in vivo*

**Figure 8:**
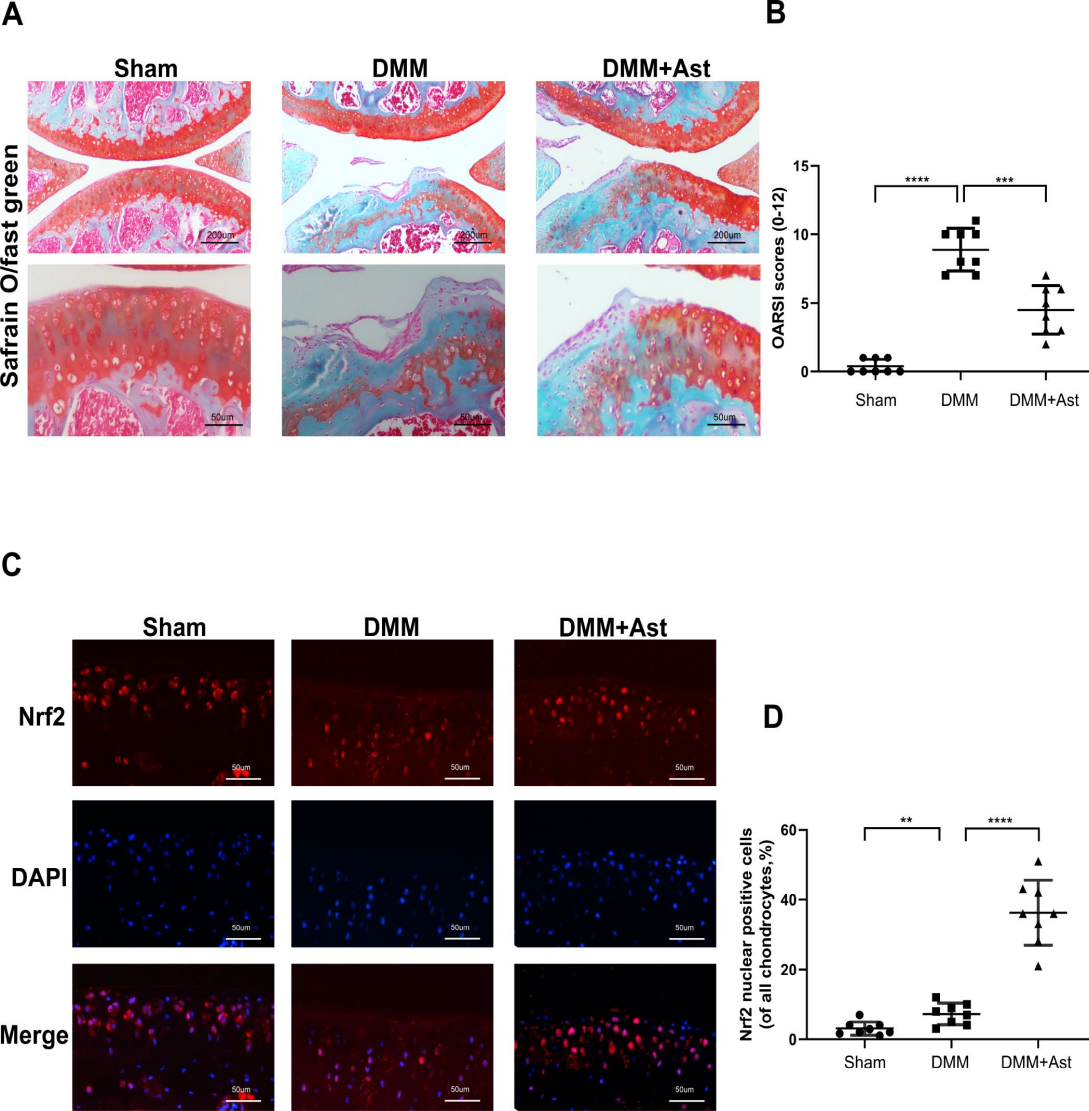
**Effects of Ast on cartilage degradation *in vivo*. Mice received intra-articular injections of Ast (20 mg/kg) or vehicle for 8 weeks after DMM surgery.** (**A**) Histological analysis of cartilage degradation was evaluated by safranin O staining. Representative safranin O staining of cartilage (scale bar: 200 μm and 50 μm). (**B**) Osteoarthritis Research Society International (OARSI) scores of three groups. (**C**) Nrf2 expression in cartilage was detected by immunofluorescence staining (scale bar: 50 μm). (**D**) Dot plots graphs represent rates of nuclear Nrf2 in total chondrocytes from each cartilage section. The data are presented as dot plots from three independent experiments. Significant differences among different groups are indicated as **P < 0.01; ***P<0.001; ****P < 0.0001.

Considering that Ast exerted prominent protective effects on OA chondrocytes, we further tested whether Ast could activate Nrf2 and attenuate OA progression in a mouse DMM model. DMM group mice were injected intra-articularly with Ast (20mg/kg) in 10 μL of solution twice a week. The other mice received 10 μL of solution only for eight weeks. OA severity was assessed by Safranin O/fast green staining, and evaluated by Osteoarthritis Research Society International (OARSI) scoring. The DMM group exhibited intact cartilage surface, obvious proteoglycan loss, and deep cartilage erosion compared to the DMM + Ast group, which was characterized by slight proteoglycan loss and erosion (Figure 8A). Also, the OARSI scores of the DMM group were significantly higher than those of the DMM + Ast group (Figure 8B). Nrf2 expression in cartilage was examined by immunofluorescence staining. In normal and OA cartilage, Nrf2 was found mainly in chondrocyte cytoplasm. By contrast, more red-stained Nrf2 was distributed in the nuclei of chondrocytes found in the cartilage of the DMM + Ast group (Figure 8C, 8D). These results suggest that Nrf2 mediates the anti-arthritic effects of Ast in experimental OA.

## DISCUSSION

Cartilage homeostasis, defined as the balance between synthesis and degradation of chondrocyte extracellular matrix, is vital to articular health. Destruction of cartilage homeostasis is the initiator and driver of OA pathology [31]. Inflammation and oxidative stress are closely related in OA progression. Inflammatory mediators such as IL-1β, TNF-α, and H_2_O_2_, (a major type of ROS) could disrupt cartilage homeostasis by causing an imbalance in cellular redox status, leading to an inflammation response, and chondrocyte apoptosis [8]. These mediators are also thought to imitate OA pathological conditions at the cellular level. Cellular redox status is primarily modulated by the transcription factor Nrf2, and Nrf2-mediated cytoprotective effects are essential for cartilage homeostasis [11]. Ast, a unique bioactive substance characterized by safety and easy access, exerts multiple functions, including anti-oxidation, anti-inflammation, and activation of Nrf2 [15, 32, 33]. In the current study, we established *in vivo* and *in vitro* OA models to investigate the anti-arthritic effects and mechanisms of action of Ast. Our findings verified that Ast can attenuate OA progression by modulating Nrf2, and inhibiting inflammation, oxidative stress, and apoptosis in OA chondrocytes.

When Nrf2 is freed from its interaction with Keap1, it enters the nucleus and regulates the expression of downstream cytoprotective genes under pathological conditions or in the presence of its activator. Studies have shown that Nrf2 activation could attenuate OA progression [34]. Absence of active Nrf2 promotes more severe cartilage damage, while Nrf2 activation reduces ECM degradation [35]. In addition, Nrf2/ARE signaling protects OA chondrocytes from oxidative and apoptotic responses [11]. In our study, Ast effectively activated the Nrf2 defense system. It blocked binding between Nrf2 and Keap1, and allowed Nrf2 to orchestrate expression of downstream defenders including HO-1 and NQO1. Ast treatment markedly maintained cartilage homeostasis of cells under stimulation with IL-1β, TNF-α, and TBHP via reduction of ADAMTS5 and MMPs, and upregulation of Collagen II. Ast significantly promoted the expression of Nrf2, HO-1, and NQO1 proteins under these pathological conditions. To verify that Nrf2 activation mediated Ast’s protective effects on cartilage homeostasis, we performed the knockdown of Nrf2 by siRNA transfection. Knockdown of Nrf2 abolished Ast-mediated inhibitory effects on the expression of ADAMTS5 and MMPs in OA chondrocytes, and simultaneously abolished Ast-mediated upregulation of Collagen II in IL-1β-stimulated chondrocytes, indicating that Nrf2 activity mediates the chondroprotective effects of Ast.

Inflammation is linked to joint symptoms, and drives pathologic changes in OA development [36]. Excessive production of the inflammatory mediators PGE2 and NO aggravates ECM degradation, and represses ECM synthesis [37]. IL-1β, present in the articular cavity of OA patients, could trigger strong inflammation responses through complex signaling pathway networks in which the MAPK signaling pathway is closely involved in IL-1β-induced inflammation. Ast is also known to notably suppress the inflammatory response via the MAPK signaling pathway in various cells [14, 38, 39]. In our study, IL-1β-induced upregulation of iNOS and COX-2 proteins was inhibited by Ast treatment. We examined the MAPK signaling pathway and found that upregulation of phosphorylated ERK and JNK by IL-1β was visibly reduced by Ast treatment. Furthermore, treatment with PD0325901, a MEK inhibitor, markedly repressed the phosphorylation of ERK, subsequently suppressed IL-1β-induced expression of COX2 and MMP3, and decreased expression of Collagen II, indirectly verifying that ERK/MAPK signaling mediates the inhibitory effects of Ast on IL-1β-induced inflammation and ECM degradation.

Many studies have reported that increased chondrocyte apoptosis correlates with aging and OA progression. Reduction of chondrocytes fails to maintain cartilage structure, and aggravates cartilage degradation [40]. Therefore, targeting the inducer of chondrocyte apoptosis, and its molecular pathways, is vital to impeding OA progression. TNF-α plays pivotal roles in controlling cell proliferation and apoptosis [5]. It is also responsible for modulation of cartilage homeostasis and chondrocyte apoptosis [41]. In our study, TNF-α not only disrupted cartilage homeostasis, it also drove chondrocytes to apoptosis by upregulating the expression of BAX and cleaved-caspase3, and downregulating Bcl-2. Ast treatment alleviated TNF-α-induced injury. Previous studies have reported that NF-κB signaling regulates chondrocyte apoptosis by affecting expression of apoptosis-related proteins [42]. Therefore, we investigated the roles of NF-κB signaling in Ast-mediated protection of chondrocytes under stimulation with TNF-α. Ast significantly blocked TNF-α-induced phosphorylation of p65 and IκBα. Furthermore, treatment with the NF-κB inhibitor QNZ inhibited phosphorylation of p65, and subsequently suppressed ECM degradation and expression of cleaved-caspase3. These results indirectly demonstrate that Ast can reduce TNF-α-induced ECM degradation and chondrocyte apoptosis by suppressing NF-κB.

Oxidative stress is another important process in chondrocyte apoptosis. It occurs due to overproduction of intracellular ROS including H_2_O_2_, superoxide anion (O^2−^) and hydroxyl radical (OH) that can cause mitochondrial damage and blockade of electron transfer, eventually resulting in apoptosis [43]. Increasing studies are reporting that excessive ROS generation is associated with cartilage degradation, and promotion of chondrocyte apoptosis. Targeting production of ROS may be an effective approach to OA treatment. Interestingly, Ast is considered to be one of the strongest anti-oxidative compounds. It reduces H_2_O_2_-induced injury in mouse Leydig cells via inhibition of ROS generation [44]. Ast also blocks H_2_O_2_-induced ROS generation, mitochondrial dysfunction, and apoptosis, in alveolar epithelial cells [45]. In this study, Ast exerted cytoprotective effects by suppressing ROS-mediated MMP collapse and apoptosis in H_2_O_2_-stimulated chondrocytes. These results are in agreement with the studies cited above. Although Ast has been demonstrated effective in safeguarding cartilage homeostasis and alleviating OA development, its clinical efficacy and appropriate dosage need to be researched in more detail.

**Figure 9:**
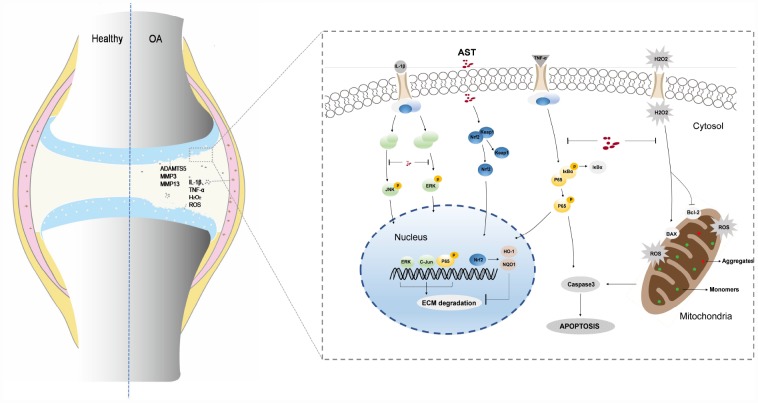
**Schematic diagram of Ast-mediated protective effects on cartilage homeostasis.** Ast attenuated ECM degradation of OA chondrocytes through the Nrf2 signaling, and ameliorated the IL-1β-induced inflammatory response and ECM degradation via blockade of MAPK signaling. In addition, Ast alleviated TNF-α-induced ECM degradation and chondrocyte apoptosis by inhibiting the NF-kB signaling, suppressed TBHP-induced oxidative stress, and subsequently reduced chondrocyte apoptosis.

In conclusion, our findings have demonstrated that Ast can maintain cartilage homeostasis through modulation of Nrf2 signaling. Ast also exerts anti-arthritic effects by inhibiting inflammation, oxidative stress, and apoptosis in mouse OA chondrocytes (Figure 9). Our results also demonstrate that Ast treatment can attenuate cartilage degradation *in vivo* by activating Nrf2. These findings provide comprehensive insights into the bioactivity of Ast in OA progression, and suggest the use of Ast for early stage clinical treatment of OA.

## MATERIALS AND METHODS

### Reagents

Astaxanthin (purity 99.54%), ERK1/2 inhibitor PD0325901, and ROS inhibitor N-acetyl-L-cysteine (NAC) were purchased from Selleck Chemicals, and were dissolved in 0.1% dimethylsulfoxide (DMSO). NF-kB inhibitor QNZ was supplied by MCE. Recombinant mouse IL-1β, and recombinant mouse TNF-α were purchased from R&D systems (Minneapolis, MN, USA). TBHP was purchased from Sigma-Aldrich (St. Louis, MO, USA). Fetal bovine serum (FBS) and Dulbecco’s modified Eagle’s medium F12 (DMEM /F12) were supplied by Gibco (NY, USA).

### Isolation and culture of murine chondrocytes

Chondrocytes were isolated from 5-day-old C57BL/6J mice (from the Laboratory Animal Center of the Tongji Medical College, Huazhong University of Science and Technology, Wuhan, China) using a previously described procedure [46]. Briefly, Cartilage was removed from the knee joints and minced, then subsequently digested with 0.25% trypsin and 0.25% collagenase. Afterwards, cells were collected and cultured in DMEM/F12 medium containing 10% FBS in a humidified atmosphere of 5% CO_2_ at 37°C. Cells were passaged at 80% confluence and transferred to a culture flask. Chondrocytes at passage 1 and 2 were used in our study.

### Cell counting Kit-8 assay (CCK-8)

Cell viability was determined using a CCK-8 assay (Boster, China). Briefly, chondrocytes were seeded into 96-well plates (5,000–10,000 cells/well) allowing adherence for 24 h and then treated with different concentrations of Ast. After 24h or 48h, 10 μl of CCK-8 was added to each well. After a one hour-reaction, absorbance was measured at 450 nm using a microplate reader (Leica Microsystems, Wetzlar, Germany).

### Reverse transcription polymerase chain reaction (RT-PCR)

RT-PCR was used to quantify the expression of related genes. The procedure was performed as previously described [47]. Total RNA was isolated from chondrocytes using a total RNA extraction kit (Toyobo, Japan). cDNA was synthesized from total RNA using a first Strand cDNA Synthesis Kit (Yeasen, China) and then amplified using SYBR Green Real-time PCR Master Mix (Yeasen, China). Each cDNA sample was assayed in triplicate. Sequences of the primers for the genes of interest are as follows: ADAMTS5 (F) 5′-GCCAGGC GGATGTGGTTCTCAA-3′, (R) 5′-ATG CGGCTCGAGTGGGCGCCCTTGT-3′. MMP3 (F) 5′-GAAACGGGACAAGTCTGTGGAG-3′, (R) 5′-ATGA AAATGAAGGGTCTTCCGGTCC-3′. MMP13 (F) 5′-GCTGGACTCCCTGTTG-3′, (R) 5′-TCGGAGCCTGT CAACT-3′. Collagen II (F) 5′-GGGAATGTCCTCTGC GATGAC-3′, (R) 5′-GAAGGGGATCTCGGGGTTG-3′. GAPDH (F) 5′-AACATCAAATGGGGTGAGGCC-3′, (R): 5′-GTTGTCATGGATGACCTTGGC-3′.

### Western blotting

Cells were washed twice with PBS and treated with RIPA lysis buffer and 1% proteinase inhibitor cocktail for 30 min on ice, followed by 30 minutes centrifugation at 12800 g. Protein concentration was measured using a bicinchoninic acid assay kit (Boster, China). Then, protein samples (25 μg) were separated on 12% SDS-PAGE gels and transferred to PVDF membranes (Millipore, Billerica, MA). Membranes were blocked with 5% bovine serum albumin dissolved in Tris-buffered saline Tween-20 (TBST) for 1 h, and subsequently incubated with primary antibodies (1:1000 - 1:300) at 4°C overnight. Collagen Type II (#15943-1-AP dilution 1:1000), BAX (#50599-2-Ig 1:1000), Nrf2 (#16396-1-AP 1:1000), HO-1 (#10701-1-AP 1:1000), cleaved-caspase-3 (19677-1-AP 1:1000) and GAPDH (60004-1-Ig 1:1000) were provided by the Proteintech Group (Wuhan, China). Antibodies against MMP13 (#ab39012 1:1000), Keap1 (#ab39012 1:1000), and NQO1 (#ab80588 1:1000) were obtained from Abcam (Cambridge, UK). Antibodies against iNOS (#sc-7271 1:1000), COX2 (#12882 1:1000), Cyclin D1 (#2978 1:1000), ERK1/2 (#4695 1:1000), Phospho-ERK (#4370 1:1000), JNK (#9258 1:1000), Phospho-JNK (#9255 1:1000), p65 (#8242 1:1000), Phospho-p65 (#3033 1:1000), IκBα (#4812 1:1000), and Phospho-IκBα (#5209 1:1000) were supplied by Cell Signaling Technology Inc. (Beverly, MA, USA). Antibodies against MMP3 (#BM4074 1:500), and ADAMTS5 (#BA3020 1:300) were purchased from Boster (Wuhan, China). An antibody against Bcl2 (#A5010 1:500) was obtained from Selleck. Membranes were then washed 3 times in TBST and incubated with a horseradish peroxidase-conjugated secondary antibody (1:5000) for 1 h. Protein bands were visualized using a western electrochemiluminescence substrate kit (Boster, China) and were analyzed with a Bio-Rad scanner (Bio-Rad, Hercules, CA). GAPDH was used as an internal control to standardize results.

### Annexin V-FITC/PI staining

An annexin V-FITC/PI kit was applied to detect apoptotic cells. After treatment, cells were collected and washed twice with cold PBS, then stained with Annexin V/PI in the dark. After 15 min, cells were analyzed using a FACS Calibur flow cytometer (BD Biosciences, USA).

### Intracellular ROS detection

ROS levels were detected using a DCFH-DA assay kit (S0033, Beyotime, China). These experiments were performed following a previously described protocol [47]. Fluorescence was assessed using a fluorescence microscope (Evos fl auto, Life Technologies, United States) and flow cytometer.

### Mitochondrial Membrane Potential (MMP) detection

Cells were seeded and cultured in 6-well plates for 24 h. After treatment as above, the MMP was examined with a JC-1 assay kit (C2006, Beyotime, China). Cells were washed with PBS and stained with the JC-1 staining solution for 20 min in darkness. Afterwards, the cells were washed with JC-1 buffer and observed under a fluorescence microscope. After treatment, cells were collected and stained with JC-1 staining solution. Red fluorescence intensity was evaluated with a flow cytometer.

### Immunofluorescence microscopy

Chondrocytes were seeded on a 24-well-plate for Nrf2 staining. After treatment, cells were fixed with 4% paraformaldehyde for 10 min at room temperature. After fixation and washing three times with PBS, cells were permeabilized with PBS containing 0.1% Triton X-100 for 10 min and blocked with 5% BSA for 1 h. Next, cells were incubated with primary antibodies against Nrf2 overnight at 4 °C. After rinsing three times with PBS, cells were incubated with Cy3-conjugated goat anti-rabbit secondary antibody for 1 h in the dark. Finally, cells were washed three times with PBS and stained with DAPI for 10 min. Images were obtained with a fluorescence microscope.

### Animals and the OA model

The Institutional Animal Care and Use Committee of Tongji Hospital (Wuhan, China) approved all animal experiments. Eight-week-old male C57BL/6 mice were supplied by the Experimental Animal Centre of Tongji Hospital. To establish an experimental OA model, the destabilized medial meniscus (DMM) surgical procedure was performed on the left knee of mice anesthetized by intraperitoneal injection of % pentobarbital (35 mg/kg) following a previously published protocol [48]. Twenty-four mice were randomly divided into three groups (n = 8 per group), the sham group, the DMM group, and the DMM+Ast group. The DMM+Ast group mice were injected intra-articularly with Ast (20 mg/kg) dissolved in 10 μL of a solution containing 20% Tween-20 twice a week for 8 consecutive weeks. The sham group and the DMM group received only 10 μL of the vehicle. Eight weeks later, joint tissue from each left knee was collected for further analysis.

### siRNA

A specific small interfering RNA (siRNA) targeting the mouse Nrf2 gene was chemically synthesized by RiboBio (Guangzhou, China) and was transfected into cells using Lipofectamine 3000 transfection reagent following the manufacturer's instructions (Thermo Fisher, UT, USA). The Nrf2 siRNA sequences are as follows: sense strand 5′-CGACAGAC CCTCCATCTA-3′.

### Histological staining and immunohistochemistry analysis

After the mice were euthanized, all left knee joints were excised and fixed in 4% paraformaldehyde for 24 h, then decalcified with 10% EDTA solution for 2 weeks. Specimens were then embedded in paraffin wax. Knee joint sections were cut to 5 μm thickness in the sagittal plane. Ten consecutive slides from every 50 μm of joint tissue were stained with safranin O/fast green to assess cartilage destruction. The severity of OA was evaluated by histopathologists using the Osteoarthritis Research Society International (OARSI) histopathology scoring system in a blinded manner [49]. After they were deparaffinized and rehydrated, sections were blocked in BSA containing 0.1% Triton X-100 for 1 h for histochemistry. Sections were then incubated with antibody to Nrf2, followed by Alexa Fluor 488 goat anti-rabbit secondary antibody to observe nuclear Nrf2 positive cells.

### Statistical analysis

All experiments were independently performed in triplicate. All data were analyzed using Graph Pad Prism 8.0 software. The results are expressed as means ± S.D. The Student’s t-test was used to compare differences between any two groups. One-way analysis of variance (ANOVA) was utilized to determine differences among groups when evaluating more than two, followed by a Tukey test. P-values < 0.05 were considered significant.
